# Supplementary material for: Characteristic and Chondrogenic Differentiation Analysis of Hybrid Hydrogels Comprised of Hyaluronic Acid Methacryloyl (HAMA), Gelatin Methacryloyl (GelMA), and the Acrylate-Functionalized Nano-Silica Crosslinker
Source: Polymers (Basel). 2022 May 13;14(10):2003. doi: 10.3390/polym14102003 (PMC9144778; doi:10.3390/polym14102003)
Supplement: Supplementary file 1 [file polymers-14-02003-s001.zip › polymers-1691101-supplementary.pdf]

Supporting Information

Characteristic and chondrogenic differentiation analysis of hybrid hydrogels comprise of hyaluronic acid methacryloyl (HAMA), gelatin methacryloyl (GelMA), and the acrylate functionalized nano-silica crosslinker

Swathi Nedunchezian[1,2,3], Che-Wei Wu[2,3], Shung-Cheng Wu[2,3], Chung-Hwan Chen[2,3,4], Je-Ken Chang[2,3,4], Chih-Kuang Wang [1,2,3,5,*]

[1] Department of Medicinal and applied Chemistry, Kaohsiung Medical University, Kaohsiung, Taiwan.

[2] Regenerative Medicine and Cell Therapy Research Center, Kaohsiung Medical University, Kaohsiung, Taiwan.

[3] Orthopedic Research Center, College of Medicine, Kaohsiung Medical University, Kaohsiung, Taiwan.

[4] Department of Orthopedics, College of Medicine, Kaohsiung Medical University, Kaohsiung, Taiwan.

[5] Graduate Institute of Medicine, Kaohsiung Medical University, Kaohsiung, Taiwan

* Corresponding author: Chih-Kuang Wang

E-mail address: ckwang@kmu.edu.tw (C.K. Wang)

Tel.: 886-7-3121101 ext. 2677; Fax: 886-7-3125339

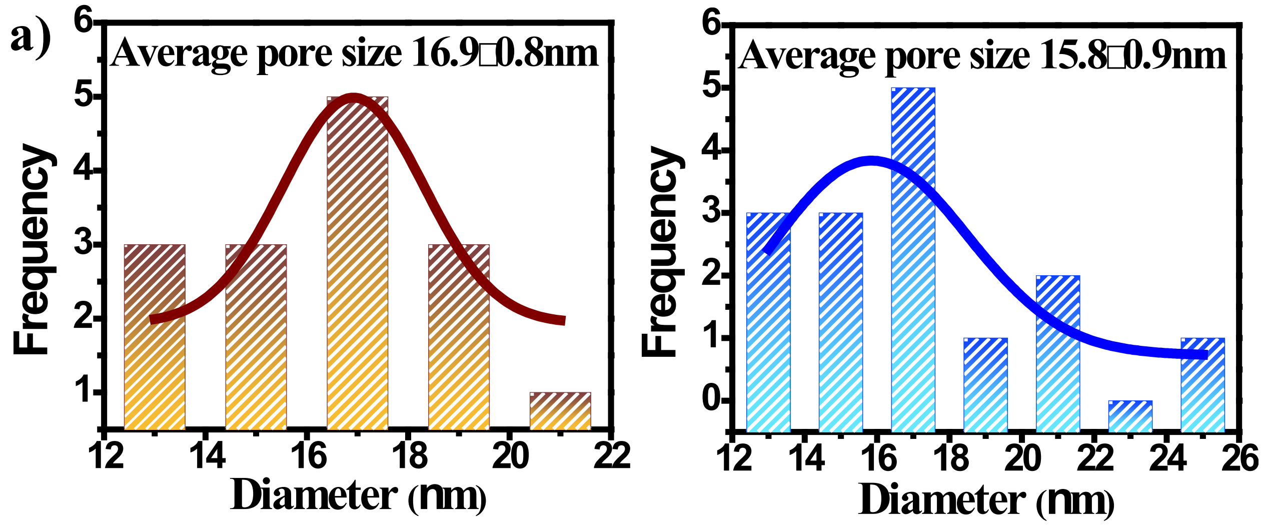

Figure S1 Particle size distribution of nano-silica (nSi) (a) and AFnSi crossliner (b).

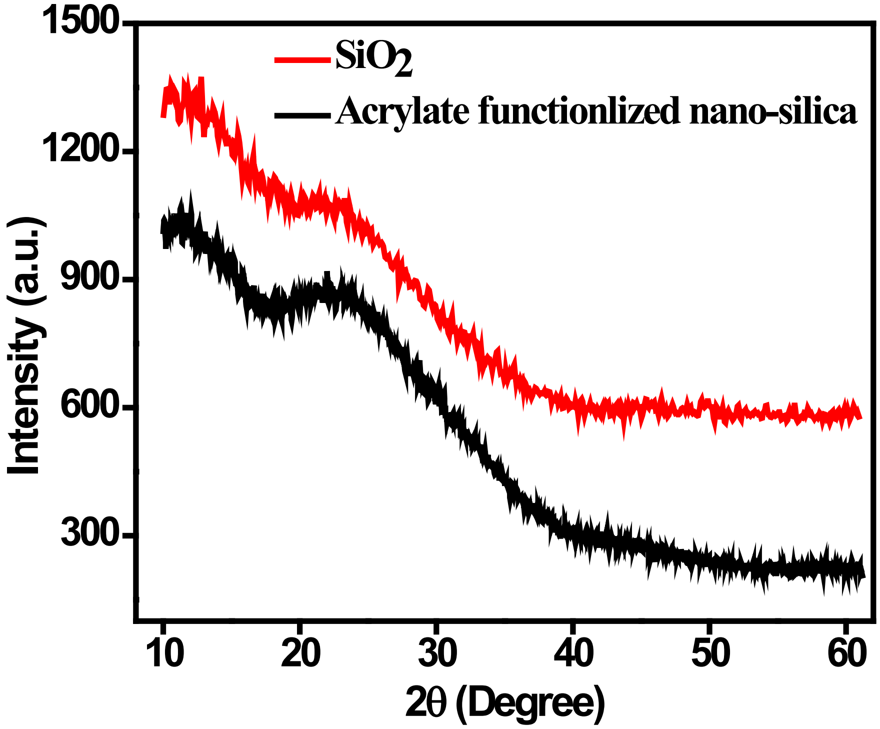

Figure S2 Demonstrates the XRD pattern of nano-silica (SiO_2) (red line) and acrylate functionalised nano-silica (black line).

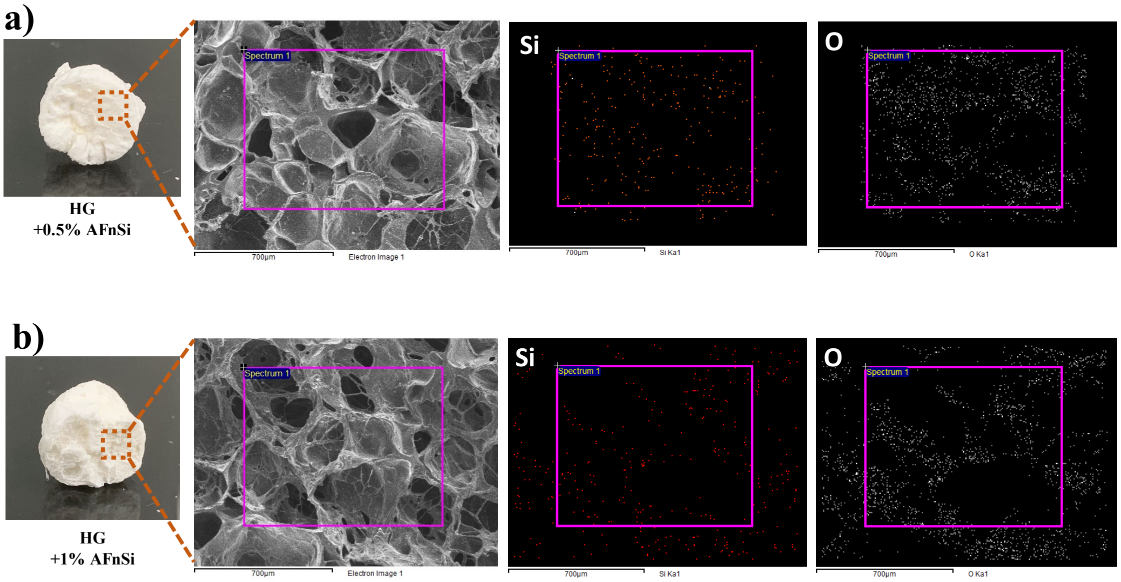

Figure S3 Side-view optical micrograph, SEM and its elemental mapping analysis of EDS image of the hybrid hydrogel HG with 0.5% AFnSi (a) and HG with 1% AFnSi crosslinker (b).

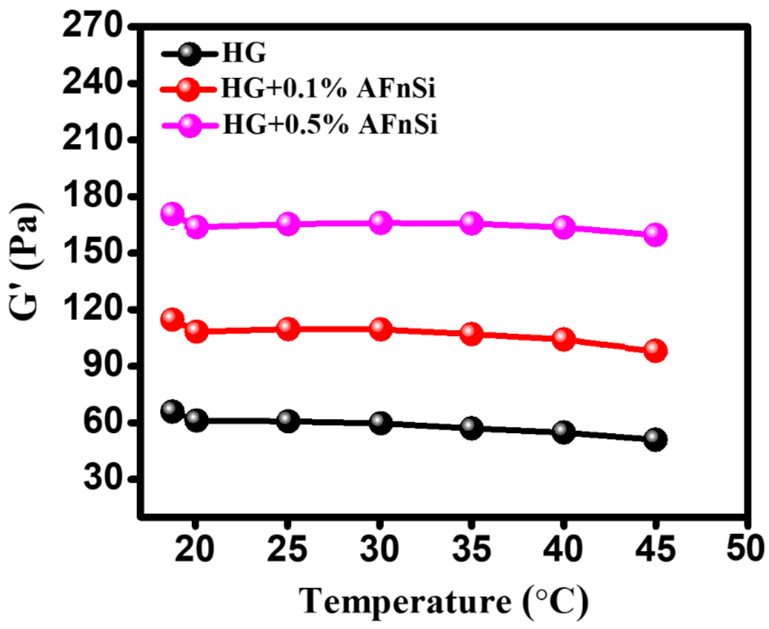

Figure S4 Storage modulus (G′) as a function of the temperature sweep for the photo-crosslinked HG hydrogel with different concentrations of AFnSi crosslinkers such as 0, 0.1, and 0.5% (w/v) respectively.
